# Small-Molecule-Mediated Depletion of the Dual-Specificity Tyrosine Phosphorylation-Regulated Kinase 1B (DYRK1B) Protein Scaffold

**DOI:** 10.1021/acsptsci.6c00238

**Published:** 2026-08-29

**Authors:** Miriam Ems, Mattias F. Lindberg, Till Adhikary, Laurent Meijer, Matthias Lauth

**Affiliations:** † Clinic of Gastroenterology, Endocrinology and Metabolism, Center for Tumor- and Immune Biology, 98594 Philipps University Marburg, 35043 Marburg, Germany; ‡ Perharidy Research Center, Perha Pharmaceuticals, 29680 Roscoff, Bretagne, France; § Institute for Biomedical Informatics and Biostatistics, Center for Tumor- and Immune Biology, Philipps University Marburg, 35043 Marburg, Germany

**Keywords:** DYRK1B, kinase inhibitor, leucettinib, metabolic disease, diabetes, NASH

## Abstract

Members of the DYRK kinase family have been implicated in the regulation of key metabolic processes. In particular, DYRK1B plays a central role in hepatic lipid metabolism and fatty liver disease. Notably, these functions depend not only on the enzyme’s catalytic activity but also on scaffolding properties. Consequently, therapeutic ambitions aiming to target the entire spectrum of DYRK1B functions must inactivate or eliminate the protein itself rather than merely inhibiting its enzymatic activity, as is achieved by current kinase inhibitors. In this study, we describe a small molecule from the leucettinib class of DYRK inhibitors (LCTB-92) that not only suppresses DYRK kinase activity but also promotes selective ablation of the DYRK1B protein while sparing the closely related paralog DYRK1A. The identification of this compound establishes a foundation for the development of selective agents capable of targeting the entire DYRK1B protein scaffold, thereby addressing catalytic-dependent as well as catalytic-independent kinase functions.

## Graphical abstract

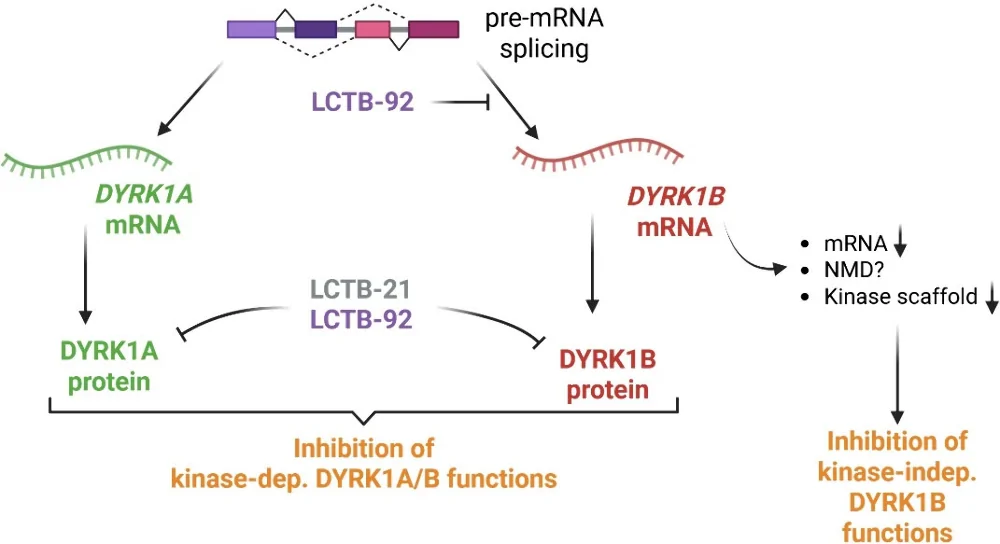

Dual-specificity tyrosine phosphorylation-regulated kinases (DYRKs) are crucial regulators of multiple processes within the cell, including the cell cycle, differentiation, signal transduction, cancer progression, and metabolic processes.^1,4–8^ A distinct subgroup within this protein family comprises the two paralogous kinases DYRK1A and DYRK1B, with the latter being particularly implicated in metabolic control. As such, mutations in the *DYRK1B* gene are associated with an inherited form of the metabolic syndrome.^9–12^ Furthermore, DYRK1B overexpression has been functionally linked to *de novo* lipogenesis in the liver and the development of nonalcoholic steatohepatitis (NASH).^13^ DYRK1B also modulates hepatic gluconeogenesis and systemic glucose homeostasis,^14,15^ making it a promising therapeutic target for several metabolic disorders. Hereby, certain DYRK1B-mediated processes rely on its kinase catalytic activity, whereas others occur independently. For instance, the FOXO1 transcription factor and hepatic gluconeogenesis are regulated through DYRK1B-mediated phosphorylation.^15^ In contrast, DYRK1B promotes hepatic lipogenesis by promoting mTOR complex 2 (mTORC2) assembly and activation in a kinase-independent manner.^13^ In addition, the key gluconeogenetic enzyme glucose-6-phosphatase is induced by DYRK1B in a kinase-independent mechanism, as is the muscle-differentiating and autophagy-promoting factor 4E-BP1.^9,16^

Consequently, effective therapeutic strategies will have to inactivate not only the enzymatic activity of DYRK1B but also its scaffolding functions in order to block the entire spectrum of effects. Although proteolysis-targeting chimeras (PROTACs) offer a potential approach,^17^ currently available compounds fail to distinguish between the closely related kinases DYRK1A and DYRK1B and degrade both proteins.

Here, we report on the characterization of a small molecule, LCTB-92, belonging to the leucettinib class of DYRK inhibitors.^18^ LCTB-92 selectively targets the DYRK1B, but not DYRK1A, protein scaffold by interfering with *DYRK1B* pre-mRNA splicing. This approach could aid in the future development of paralogue-specific DYRK inhibitors blocking not only the kinase activity but also ablating the protein’s scaffolding function.

During our studies of DYRK kinases, we serendipitously observed that an adamantane moiety-containing member of the Leucettinib (LCTB) class of pharmacological DYRK inhibitors (i.e., LCTB-92)^18^ strongly suppressed endogenous DYRK1B protein levels in human Panc1 pancreatic cancer (pancreatic ductal adenocarcinoma; PDAC) cells (Figure 1A,B). To our surprise, the closely related DYRK1A protein was unaffected by this compound (Figure 1A,C). As experimental controls, we applied either LCTB-21 (another LCTB-class DYRK inhibitor, which is now in clinical phase I testing^19^) or the chemical isomers (iso-LCTBs) of LCTB-21 and LCTB-92 (for chemical structures, see Figure S1A), which are both inactive and cannot block DYRK enzyme activity. In contrast to LCTB-92, none of these control compounds resulted in gross reduction of DYRK1B in treated cells (Figure 1A,B). These findings were not limited to Panc1 cells and could also be detected in the human PDAC cell line PaTu 8988T (Figure 1D–F) and in other human (Figure S1B,C) and mouse cell lines (Figure S1D,E), demonstrating the generality of the observation. Of note, other commercially available DYRK inhibitors (such as harmine,^20^ AZ191,^21^ and Indy^22^) used at concentrations known to block DYRK kinase activity did not abrogate DYRK1B levels in treated cells (Figure 1G,H).

**Figure 1:**
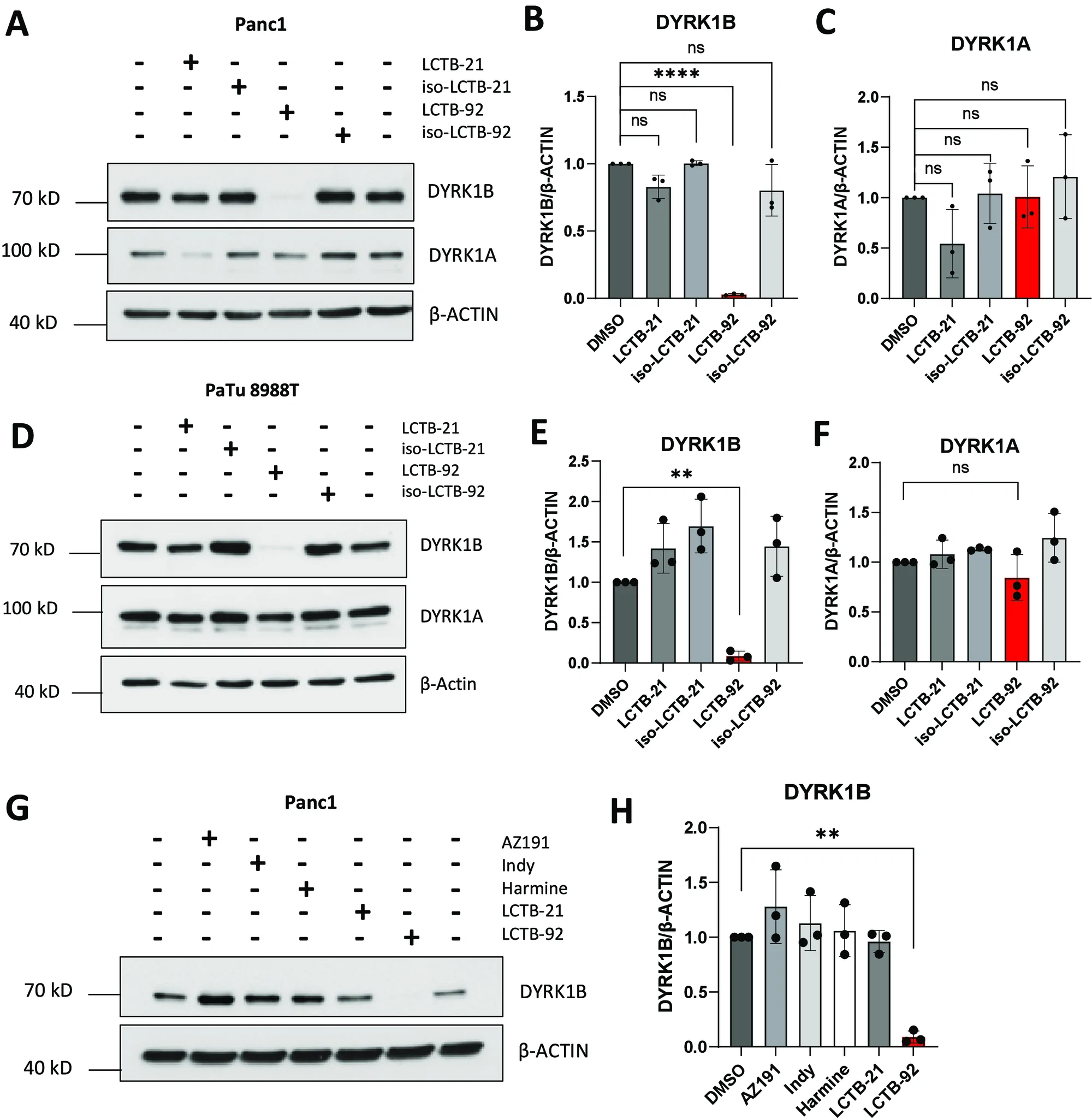
**LCTB-92 treatment decreases endogenous DYRK1B protein levels.** (A) Western blot of Panc1 lysates upon 24 h treatment with 1 μM LCTB-21, iso-LCTB-21, LCTB-92, iso-LCTB-92, or the vehicle (DMSO). Shown is one representative of *n* = 3. (B) Quantification of DYRK1B protein abundance in 24 h-treated Panc1 cells (1 μM compound). (C) Quantification of DYRK1A protein abundance in 24 h-treated Panc1 cells (1 μM compound). (D) Western blot of PaTu 8988T lysates upon 24 h treatment with the indicated compounds at 1 μM. Shown is one representative of *n* = 3. (E) Quantification of DYRK1B protein abundance in 24 h-treated PaTu 8988T cells (1 μM compound). (F) Quantification of DYRK1A protein abundance in 24 h-treated PaTu 8988T cells (1 μM compound). (G) DYRK1B protein levels in Panc1 cells treated with the indicated DYRK inhibitors at a concentration of 1 μM for 24 h. Shown is one representative of *n* = 3. (H) Quantification of DYRK1B protein abundance in 24 h-treated Panc1 cells (1 μM compound).

Next, we investigated the concentration dependency of the observed LCTB-92-mediated effect. As can be seen in Figure 2A,B, LCTB-92 showed efficacy on DYRK1B protein levels already at 0.1 μM and displayed an IC_50_ value of around 0.7 μM (Figure 2B). High concentrations of 10 μM showed unspecific effects, which were probably caused by cellular toxicity, limiting its use at very high doses. Again, LCTB-21 did not display any dose-dependent impact on DYRK1B (Figure 2C,D). Moreover, the modulation of DYRK1B levels by 1 μM LCTB-92 was not immediate and required around 4 h to reach 50% reduction, and around 8–16 h to reach profound effects (Figure 2E,F), arguing for an indirect mode of action of this compound. In line with previous results, the control molecule LCTB-21 did not modulate DYRK1B over this time period (Figure 2G,H).

**Figure 2:**
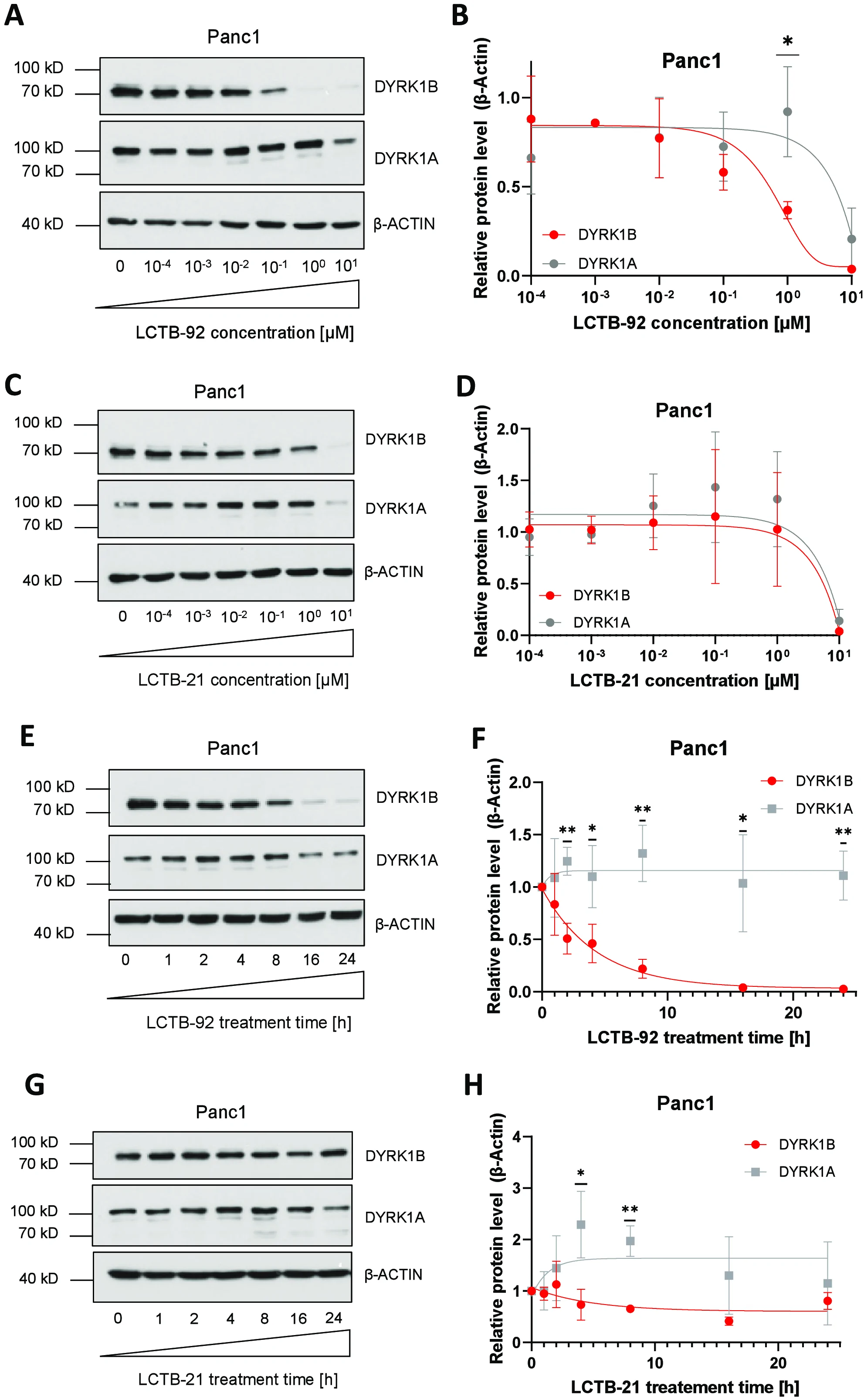
**Concentration- and time-dependent decrease of DYRK1B but not DYRK1A levels.** (A) DYRK1A and DYRK1B protein levels in 24 h-treated Panc1 cells. Shown is one representative of *n* = 3. (B) Quantification of DYRK protein levels upon increasing concentration of LCTB-92, relative to vehicle control (0 μM compound). Shown is the mean of *n* = 3 independent experiments ± SD (two-tailed unpaired *t* test). (C) DYRK1A and DYRK1B protein levels in 24 h-treated Panc1 cells. Shown is one representative of *n* = 3. (D) Quantification of DYRK protein levels upon increasing concentration of LCTB-21, relative to vehicle control (0 μM compound). Shown is the mean of three independent experiments ± SD (two-tailed unpaired *t* test). (E) DYRK protein levels in 24 h-treated Panc1 cells over time (1 μM compound). Shown is one representative of *n* = 3. (F) Quantification of DYRK protein levels over time (1 μM compound), relative to vehicle control (0 μM compound). Shown is the mean of *n* = 3 independent experiments ± SD (two-tailed unpaired *t* test). (G) DYRK protein levels in 24 h-treated Panc1 cells over time (1 μM compound). Shown is one representative of *n* = 3. (H) Quantification of DYRK protein levels over time (1 μM compound), relative to vehicle control (0 μM compound). Shown is the mean of three independent experiments ± SD (two-tailed unpaired *t* test).

To our surprise, the protein stability of endogenous DYRK1B was unperturbed by LCTB-92, as assessed by cycloheximide (CHX) chase experiments, in which *de novo* protein synthesis is blocked and the stability (and/or decay) of existing protein is investigated (Figure S2A). Along these lines of research, cotreatment of LCTB-92 with inhibitors of degradative cellular structures such as the proteasome (bortezomib), autophagosomes (bafilomycin, 3-MA), or lysosomes (chloroquine), did not rescue LCTB-92-suppressed DYRK1B protein levels (Figure S2B). Taken together, these data suggest that LCTB-92 does neither impinge on DYRK1B’s protein stability, nor does it promote its degradation.

We therefore analyzed the levels of the *DYRK1B* mRNA transcript in human Panc1 cells and found strongly reduced *DYRK1B* mRNA levels in LCTB-92-exposed cells (Figure 3A), with *DYRK1A* mRNA not being affected (Figure S3A). Similar findings were made in additional human and mouse cells (Figure S3B,C). Moreover, none of the related transcripts *DYRK2*, *DYRK3*, and *DYRK4* were modulated by LCTB-92 (Figure S3D-F), supporting selectivity for *DYRK1B*.

**Figure 3:**
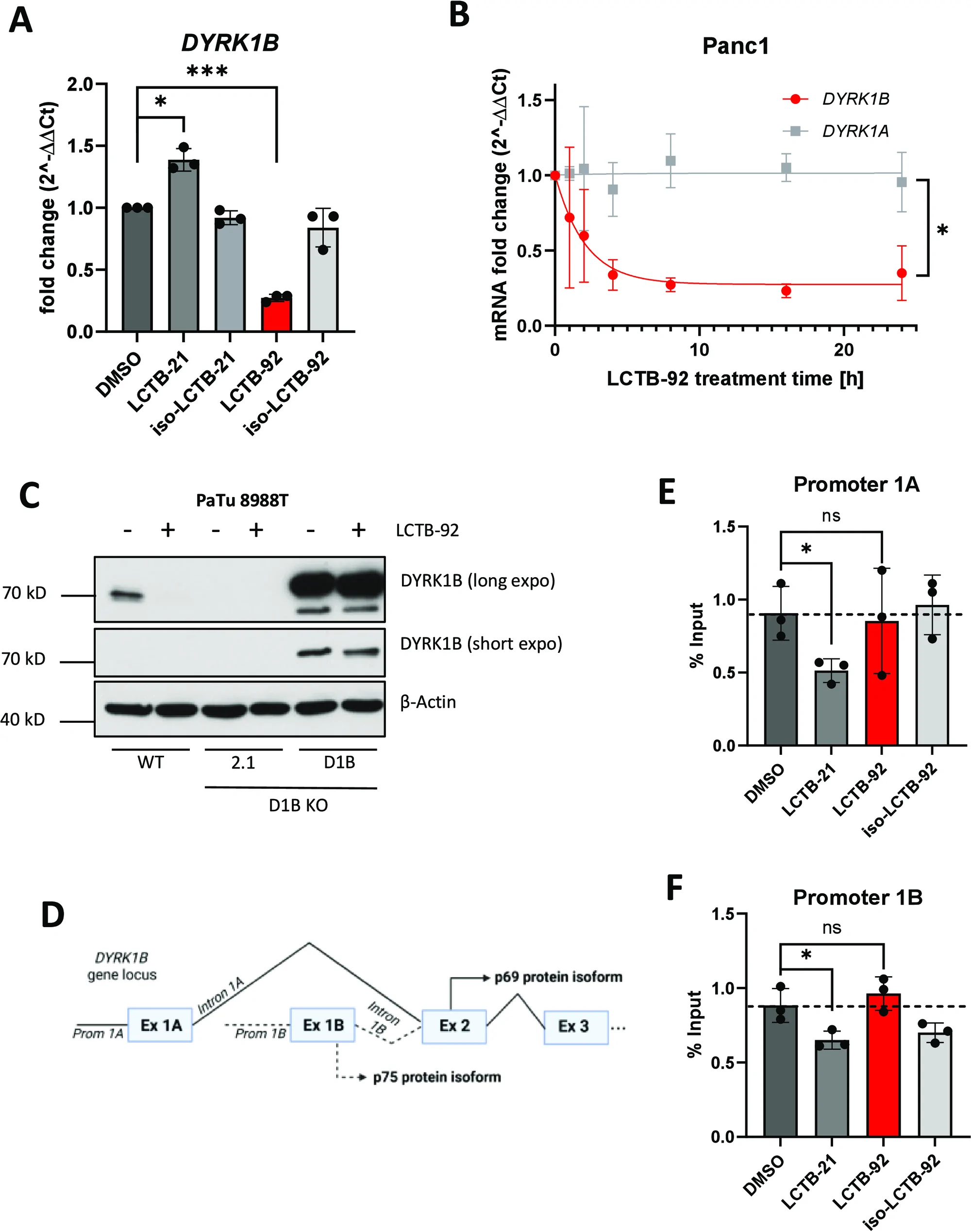
**LCTB-92 treatment decreases *DYRK1B* mRNA levels.** (A) Quantitative PCR detecting *DYRK1B* levels in Panc1 cells treated with the indicated compounds (1 μM for 24 h). Shown are the mean ± SD of three individual experiments. (B) Quantification of DYRK1*A*/1B mRNA over time in treated Panc1 cells (1 μM compound). The graph shows the results of *n* = 3 independent experiments (mean ± SD; two-tailed unpaired *t* test). (C) Western blot of PaTu 8988T lysates after 24 h treatment with 1 μM LCTB-92 or DMSO. WT = wild-type cells; D1B KO = DYRK1B KO (clone 2.1); D1B KO/D1B = rescue of KO. Shown is one representative of *n* = 3. (D) Graphical depiction of the human *DYRK1B* gene locus and its associated transcripts. (E) ChIP-qPCR of RNA-Polymerase II (RNAPII) binding to the promoter 1A of the human *DYRK1B* gene in Panc1 cells. Shown is the mean ± SD of one representative experiment measured in triplicate of *n* = 2 (two-tailed unpaired *t* test). Treatment: 1 μM compound for 16 h. (F) ChIP-qPCR of RNA-Polymerase II (RNAPII) binding to the promoter 1B of the human *DYRK1B* gene in Panc1 cells. Shown is the mean ± SD of one representative experiment measured in triplicate of *n* = 2 (two-tailed unpaired *t* test). Treatment: 1 μM compound for 16 h.

Subsequent kinetic studies revealed a maximum reduction of *DYRK1B* mRNA after 4 h of treatment (Figure 3B), possibly explaining why changes in protein levels were not immediately seen after drug addition. Unexpectedly, however, *DYRK1B* mRNA stability and decay kinetics were identical between solvent and LCTB-92-treated conditions (Figure S3G,H), ruling out a functional role for LCTB-92 in *DYRK1B* mRNA turnover. We therefore went on to decipher the cellular generation of this transcript by regulated gene transcription. In order to address the relevance of the endogenous gene promoter for the LCTB-92 effect, we first removed endogenous DYRK1B protein in human PaTu 8988T cells by CRISPR/Cas9, and then rescued it by reintroducing plasmid-driven DYRK1B. The latter construct harbored the unrelated promoter region of the human elongation factor 1α (*EF1α*) gene. Hence, *DYRK1B* transcription was no longer controlled by elements from the endogenous *DYRK1B* gene promoter. As expected, LCTB-92 treatment eliminated endogenous DYRK1B protein in wild-type cells, but was surprisingly unable to grossly impact plasmid-derived DYRK1B (Figure 3C). These results hinted toward a possible necessity for the endogenous promoter mediating the effects of LCTB-92. In order to address this issue in more detail, we performed chromatin immunoprecipitation (ChIP) experiments using an antibody directed against endogenous RNA-polymerase II (RNA-Pol II) as a surrogate for locus-specific active gene transcription. The human *DYRK1B* locus contains two promoter regions (promoter 1A and promoter 1B) driving the transcription of two 5′ exons (exon 1A and exon 1B; see Figure 3D). Transcription from promoter 1A leads to generation of the predominant isoform p69 as well as the less frequent p66 isoform. Initiation from promoter 1B instead yields a transcript encoding the isoform p75.^23^ However, neither the promoter region upstream of exon 1A nor the 1B promoter region showed distinct changes in RNA-Pol II binding upon LCTB-92 exposure (Figures 3E,F and S3I). This result implies that LCTB-92 does not affect *DYRK1B* mRNA transcription through regulation of its transcription and also disfavors related processes such as chromatin remodeling and/or epigenetic promoter modulation.

The fact that LCTB-92 neither modulated the transcription from the endogenous *DYRK1B* promoter (Figure 3E,F), nor from an unrelated (EF1α) one (Figure 3C), led us to the possibility of RNA splicing as a putative underlying mechanism. In line with our previous results, PCR primer pairs (Figure 4A) detecting the correctly spliced p69 transcript revealed a significant downregulation upon LCTB-92 treatment (Figure 4B). In contrast, the levels of an incompletely spliced p69 transcript (still containing the intronic sequence downstream of exon 1A (“intron 1A” in Figure 3D)) were clearly increased by LCTB-92 (Figure 4C). Comparable results were obtained with the very weakly expressed p75 transcript, where the correctly spliced isoform was also reduced by LCTB-92 (Figure 4D), and the unspliced p75 showed slightly increased levels (Figure 4E). Taken together, these findings strongly argue for LCTB-92 impinging on mechanisms of (alternative) splicing. In fact, LCTB-92-mediated retention of intron 1A of the predominant p69 isoform would introduce two novel upstream ATG start codons into the transcript (Figure 4F). As exon 1A is lacking any ATG codons, it can be assumed that particularly the first of the new start codons would be used. In that case, the translation machinery would run into a STOP codon shortly after, abandoning production of the DYRK1B protein. Even if the second START codon were to be used, it would have the wrong reading frame and translation would also terminate in exon 2 (Figure 4F). Thus, LCTB-92 interferes with correct splicing of intron 1*A*/1B, resulting in loss of protein production. Concomitantly, the DYRK1B kinase-independent function of mTORC2 activation is suppressed, as evidenced by decreased levels of the direct mTORC2 phosphorylation target AKT^S473^ (Figure S3J).

**Figure 4:**
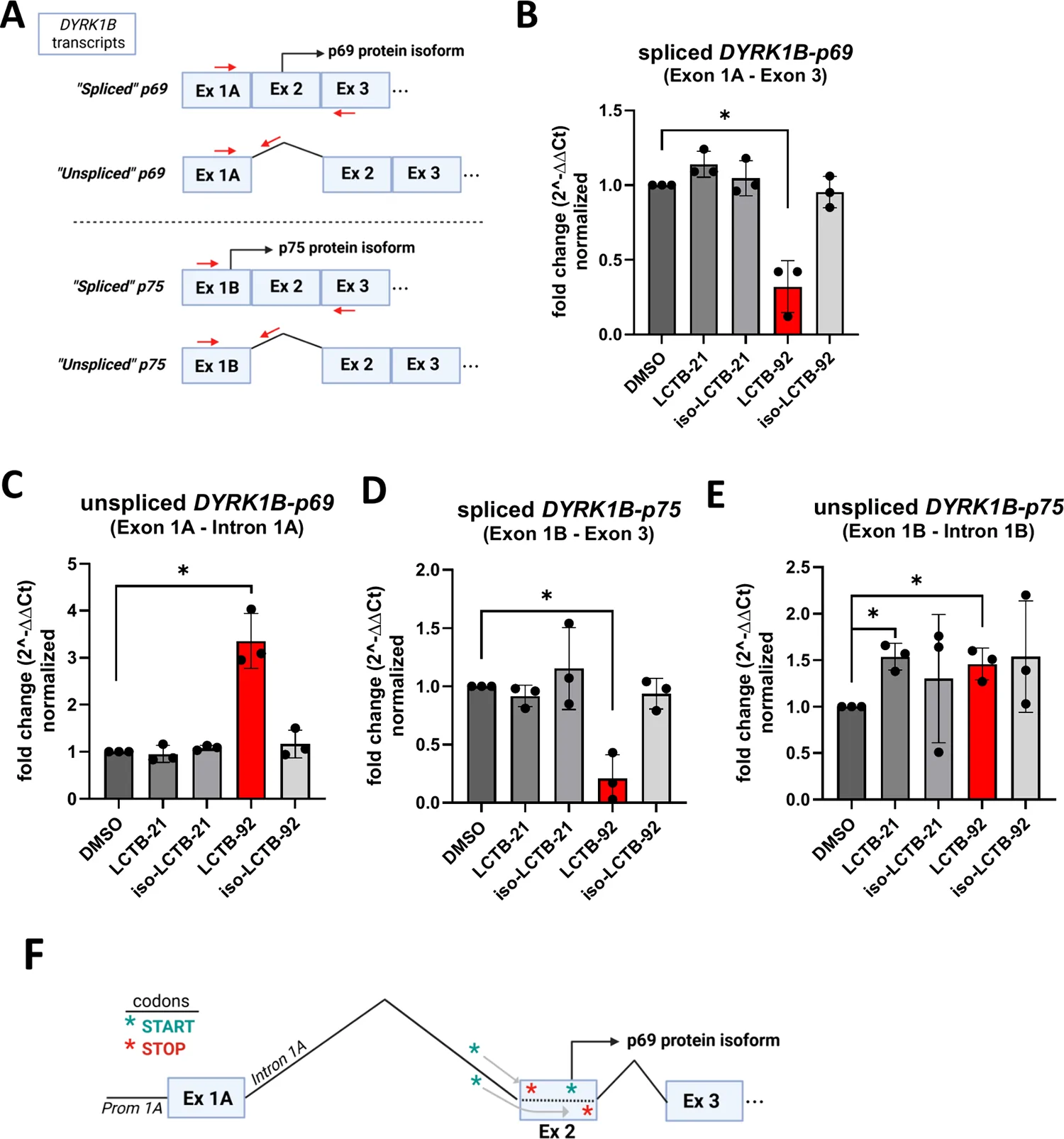
**LCTB-92 impacts *DYRK1B* mRNA splicing.** (A) Graphical scheme of the human *DYRK1B* isoforms p69 and p75 and the primer pairs (red arrows) used in the study to distinguish correctly spliced from unspliced variants. (B) Quantification of correctly spliced *DYRK1B-p69* transcript (normalized to total *DYRK1B* mRNA levels) in Panc1 cells (1 μM compound for 24 h). Shown is the mean ± SD of *n* = 3 independent experiments. (C) Quantification of nonspliced (intron-retaining) *DYRK1B-p69* transcript (normalized to total *DYRK1B* mRNA levels) in Panc1 cells (1 μM compound for 24 h). Shown is the mean ± SD of three independent experiments. (D) Quantification of correctly spliced *DYRK1B-p75* transcript (normalized to total *DYRK1B* mRNA levels) in Panc1 cells (1 μM compound for 24 h). Shown is the mean ± SD of three independent experiments. (E) Quantification of nonspliced (intron-retaining) *DYRK1B-p75* transcript (normalized to total *DYRK1B* mRNA levels) in Panc1 cells (1 μM compound for 24 h). Shown is the mean ± SD of *n* = 3 independent experiments. (F) Graphical depiction of the predominant human *DYRK1B-p69* splicing and the new upstream START/STOP codons being introduced by LCTB-92-mediated aberrant splicing.

In general, we could show that the small molecule compound LCTB-92 exerts interesting and very specific effects on DYRK1 kinases: while treatment with this molecule blocks the enzymatic function of both DYRK1A and DYRK1B,^18^ it only abrogates the protein scaffold of DYRK1B. This specificity is due to its effects on *DYRK1B* mRNA splicing. While the exact mechanism on how LCTB-92 impacts the splicing machinery remains to be elucidated, it explains why LCTB-92 does not affect plasmid-derived (and thus intronless) DYRK1B. With respect to DYRK1B, the final effect is a transcript with premature termination codons, potentially resulting in degradation of the mRNA by nonsense-mediated decay (NMD).^24^

The reasons for the DYRK1B selectivity of LCTB-92 are currently unknown. Both LCTB compounds inhibit DYRK1A and DYRK1B with comparable potency (IC_50_ LCTB-21 for DYRK1A/DYRK1B: 2.4/6.7 nM; IC_50_ LCTB-92 for DYRK1A/DYRK1B: 1.2/1.8 nM^19,25^ ). However, given that DYRKs are related to the splicing-regulating CLK kinases, it can be hypothesized that LCTB-92 might possess a particular ability to affect some of these kinases and their associated splicing factors and transcripts. With respect to kinase inhibition, LCTB-92 and LCTB-21 display some selectivity for DYRK1*A*/1B over DYRK2/3/4, but both inhibit CLKs at nanomolar concentrations,^18^ arguing against a simple explanation due to CLK inhibition. Of note, inhibiting general splicing with Isoginkgetin reduces both *DYRK1A* and *DYRK1B* mRNA levels (not shown). Thus, LCTB-92 displays an interesting specificity that requires further investigation. In any case, investigating this mechanism in more depth may allow future approaches for the selective removal of defined paralogues. In the specific case of DYRK1B, this might be of high value in the setting of metabolic disease, where certain effects are mediated by non-enzymatic functions.

## Materials and Methods

### Cell Culture

The general culture of all cell lines was as described in ref 1. Panc1 and PaTu 8988T were obtained from CLS and DSMZ, respectively. HEK293T and Ovcar3 cells were purchased from ATCC. The mKPC4 tumor cell line was generated from a murine Kras^G12D/+^; p53^R172H/+^; Pdx1-Cre (KPC) pancreatic tumor.^1^ Mouse 3T3-L1 preadipocytes were a kind gift of Kerstin Stemmer (Univ. Augsburg). In all experiments, culture medium was changed to starving conditions (DMEM (high glucose) supplemented with 0.5% FBS and 1% Pen/Strep) during the course of the experiment (i.e., from treatment initiation until cell harvesting). If not indicated otherwise, cells were treated with a compound concentration of 1 μM for 24 h. The following DYRK inhibitors were used: AZ191 (Biomol), Indy (Biomol), Harmine (Sigma), LCTB-21, LCTB-92, and their inactive isoforms iso-LCTB-21 and iso-LCTB-92 (all LCTBs from Perha Pharmaceuticals).

### Generation of KO and Rescue Cell Lines

The procedure of generating CRISPR-mediated knockout of DYRK1B can be found in ref 1. The 20 bp CRISPR guide sequence was as follows: human *DYRK1B* (CRISPR#2): GGT­TGT­CGT­CAT­CATA­ACCA. The rescue of DYRK1B KO cells (clone 2.1) was conducted by transfecting cells with the EF-DYRK1B plasmid^2^ using the Helix-In transfection reagent (Oz Biosciences, OZB-HX10500). After 48 h of incubation, cells were selected using blasticidin S (Thermo Fisher Scientific) until single clones could be collected (clone 3 was used in the case of 1B.WT-rescue, and clone 9 in the case of 1B.YF-rescue).

### Western Blotting

Samples for Western blotting were collected by lysis of cells in 1× Laemmli buffer. Subsequently, lysates were separated by SDS-PAGE. Afterward, the gels were blotted on Immobilon–polyvinylidene difluoride (PVDF) membranes (Millipore). After blocking with skimmed milk, membranes were incubated with primary antibodies (α-DYRK1B antibody (Cell Signaling Technology D40D1, #5672); α-DYRK1A antibody (Cell Signaling Technology, #2771); α-β-actin antibody (Sigma-Aldrich AC-15, A5441); α-phospho-AKT^S473^ (Cell Signaling Technology D9E, #4060)). Subsequently, membranes were incubated with the corresponding secondary antibody, which is linked to a horseradish peroxidase (α-mouse IgG, HRP-linked antibody (Cell Signaling Technology, #7076), α-rabbit IgG, HRP-linked antibody (Cell Signaling Technology, #7074)). To detect HRP signal, Pierce ECL Western Blotting Substrate (Thermo Scientific) was used according to the manufacturer’s protocol.

### Chromatin Immunoprecipitation (ChIP)-qPCR

For ChIP-qPCR, fixation of confluent cells was performed with 1% formaldehyde in culture medium for 10 min at room temperature followed by quenching with 125 mM glycine for 5 min. Afterward, ChIP was performed essentially as described previously,^3^ using 4 μg per sample of the following antibodies: IgG pool (Sigma-Aldrich Cat #I5006 RRID:AB 1163659), α-RPB1 CTD (RNA polymerase II, RRID AB_2565554, BioLegend #664906); and protein A-Sepharose (GE Healthcare Life Sciences, #1752800) for precipitation. An input sample representing 1% of the chromatin used per IP was reverted in parallel. Samples were purified using the Qiagen PCR purification kit according to the manufacturer’s instructions. Afterward, quantitative PCR was performed using the following primers: Promoter A (forward: TAC­CCA­GTA­ATG­CAA­CCAG, reverse: GAG­TGT­CAG­TGT­GGG­TCA), Promoter B (forward: TCA­GCC­AAT­CAC­AGCC­TAGG, reverse: GTT­GAA­TTC­CTG­GAAC­CGGC).

### Quantitative PCR (qRT-PCR)

For quantitative PCR (qRT-PCR), samples were collected for RNA isolation, using the NucleoSpin RNA isolation kit (Marchery-Nagel, Düren, Germany). RNA was isolated following manufacturer’s protocol. Afterward, cDNA was synthesized, using the ABScript II cDNA First-Strand Synthesis Kit with Oligo d­(T)_23_VN primers (ABclonal, Düsseldorf, Germany). Quantitative PCR was conducted using the 2× Universal SYBR Green FASt qPCR Mix (ABclonal, Düsseldorf, Germany), Mic Tubes and Caps (bio molecular systems, Australia) and the Q-qPCR instrument (Quantabio, Beverly, MA). C_T_ values of genes of interest were normalized to the levels of human *RPLP0*. Afterward, treatment conditions were normalized on vehicle control. The following human primer pairs were used: *RPLP0* (forward: CCT­TCT­CCT­TTG­GGC­TGG­TCA­TCCA; reverse: CAG­ACA­CTG­GCA­ACA­TTG­CGG­ACAC), *DYRK1A* (forward: GGA­AAA­CGG­GAG­TAC­AAA­CCA­CCA­GGA; reverse: GAC­CGT­ATG­ACC­TGA­CTC­CCC­AGCA), *DYRK1B* (forward: TTG­GCC­AGG­TGG­TGA­AAG­CCT­ATGA (exon 4/5); reverse: CAA­TCT­GGG­CCT­GGT­TCA­GGA­AAGC (exon 5)) (total abundance measurements), *DYRK2* (forward: CAG­CGG­CCA­TGT­TAA­CCA­GGA­AACC; reverse: GCC­GAC­ATG­CAG­GTG­ATC­ATT­CATT), *DYRK3* (forward: TGG­TGG­TCC­CAA­TAA­TGG­AGG­GTA­TGA; reverse: CTG­CCC­AAA­ACT­CCC­CTT­GCC­AATA), *DYRK4* (forward: GGA­CCT­CAC­GAT­GGT­GCT­GAA­AACC; reverse: GCT­GTG­GCT­TGA­GGT­TCC­GAG­ACTG). The following mouse qPCR primers were used: *Dyrk1a* (forward: CAG­CTC­TTG­TCA­GTT­GGG­GCA­GAGG; reverse: TCC­AAG­GGA­CCA­CAT­GTC­GAT­AGCA), Dyrk1b (forward: TTG­ACA­CCT­GCC­CCT­CCT­CTA­GCAC; reverse: GGC­CCC­CAC­AAT­ATC­GGT­TGC­TGTA), *Rplp0* (forward: TGC­ACT­CTC­GCT­TTC­TGG­AGG­GTGT; reverse: AAT­GCA­GAT­GGA­TCA­GCC­AGG­AAGG).

For isoform-specific questions, the following primer pairs were used: spliced *DYRK1B-p69* (forward: ATA­CCC­TCA­GCC­AGT­GGG­CAG­GTCT (exon 1A); reverse: GGC­AGT­AGC­CGC­ACA­TCA­GGCA­ATAC (exon 3)); unspliced *DYRK1B-p69* (forward: ATA­CCC­TCA­GCC­AGT­GGG­CAG­GTCT (exon 1A); reverse: TCC­TCC­CTT­GGC­CTC­GTG­CTA­AGTC (intron 1A)); spliced *DYRK1B-p75* (forward: TCC­CCC­GTC­CAC­CTG­CTC­TAC­TCTT (exon 1B); reverse: GGC­AGT­AGC­CGC­ACA­TCA­GGCA­ATAC (exon 3)); unspliced *DYRK1B-p75* (forward: TCC­CCC­GTC­CAC­CTG­CTC­TAC­TCTT (exon 1B); reverse: CCT­CCC­ACA­CCA­GGA­TCA­TCT­CTCC (intron 1B)). p69 and p75 transcripts were first normalized to *RPLP0* (see above for primers), followed by normalization to total *DYRK1B* transcript (forward: GCC­TCC­TGA­TGA­CCC­TGC­CAC­TCT; reverse: CAG­ATG­GGG­GAG­GGT­ATG­GCT­TCAG (both exon 11)).

### Statistical Analysis

If not otherwise stated, statistical analysis was performed with two-tailed one-sample (unpaired) *t* test, *p* < 0.05 *, *p* < 0.01 **, *p* < 0.001 ***, *p* < 0.0001 ****. Figures were prepared with Graph Pad Prism and BioRender.

## Supplementary Material



## Abbreviations

DYRK: dual-specificity tyrosine phosphorylation-regulated kinase
LCTB: leucettinib
PDAC: pancreatic ductal adenocarcinoma
WT: wild-type
KO: knockout
WB: Western blot
