# Supplementary material for: Small-Molecule-Mediated Depletion of the Dual-Specificity Tyrosine Phosphorylation-Regulated Kinase 1B (DYRK1B) Protein Scaffold
Source: ACS Pharmacol Transl Sci. 2026 Aug 29;9(9):2419–25. doi: 10.1021/acsptsci.6c00238 (PMC13632892; doi:10.1021/acsptsci.6c00238)
Supplement: Supplementary file 1 [file pt6c00238_si_001.pdf]

Supporting information

Small molecule-mediated depletion of the Dual Specificity Tyrosine Phosphorylation-Regulated Kinase 1B (DYRK1B) protein scaffold

Miriam Ems[1], Mattias F. Lindberg[2], Till Adhikary[3], Laurent Meijer[2], Matthias Lauth[1,*]

[1] Philipps University Marburg; Clinic of Gastroenterology, Endocrinology and Metabolism; Center for Tumor- and Immune Biology, 35043 Marburg, Germany.

[2] Perharidy Research Center, Perha Pharmaceuticals, 29680 Roscoff, Bretagne, France.

[3] Philipps University Marburg; Center for Tumor- and Immune Biology, Institute for Biomedical Informatics and Biostatistics, 35043 Marburg, Germany.

[*] **Corresponding author**: Matthias Lauth, Philipps University Marburg, Center for Tumor- and Immune Biology, Hans-Meerwein-Str. 3, 35043 Marburg, Germany. Email: lauth@staff.uni-marburg.de

Figure S1: LCTB-92 reduces endogenous DYRK1B protein levels in several human and mouse cell lines.

Figure S2: LCTB-92 does not affect DYRK1B protein stability.

Figure S3: LCTB-92 does not affect *DYRK1B* mRNA stability.

Figure S4: Selected additional Western blots for verification.

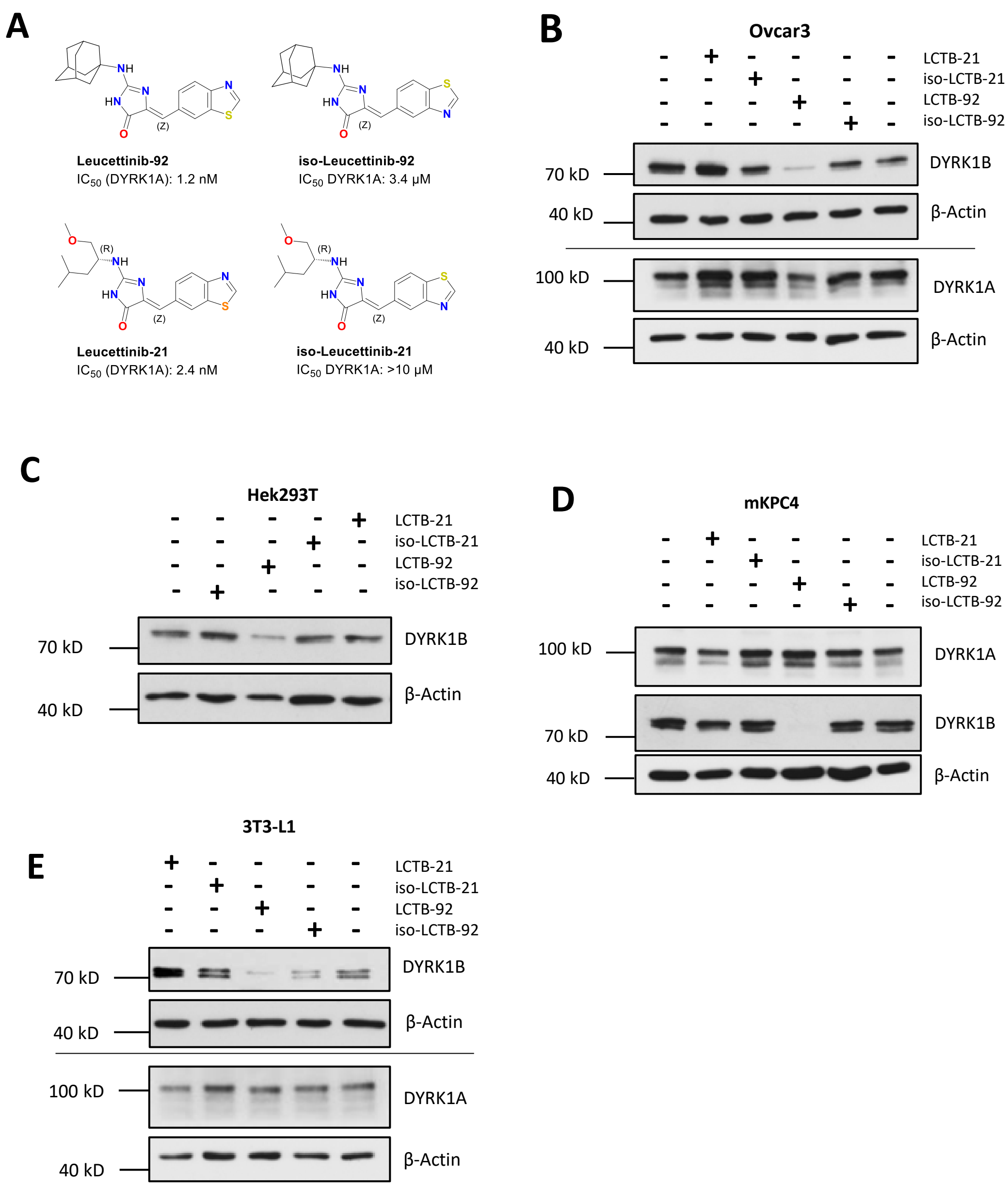

Figure S1. LCTB-92 reduces endogenous DYRK1B protein levels in several human and mouse cell lines.

A Chemical structures of LCTB-21, LCTB-92, and their corresponding inactive iso-controls. *In vitro* IC_{50} values for DYRK1A are also given for comparison. **B** DYRK1A and DYRK1B protein levels in Ovcar3 human ovarian carcinoma cells. Shown is one representative of n = 2. **C** DYRK1B protein levels in Hek293T human embryonic kidney cells. Shown is one representative of n = 2. **D** DYRK1A and DYRK1B protein levels in mKPC4 mouse PDAC cells. Shown is one representative of n = 2. **E** DYRK1A and DYRK1B protein levels in undifferentiated 3T3-L1 mouse pre-adipocytes. Shown is one representative of n = 2.

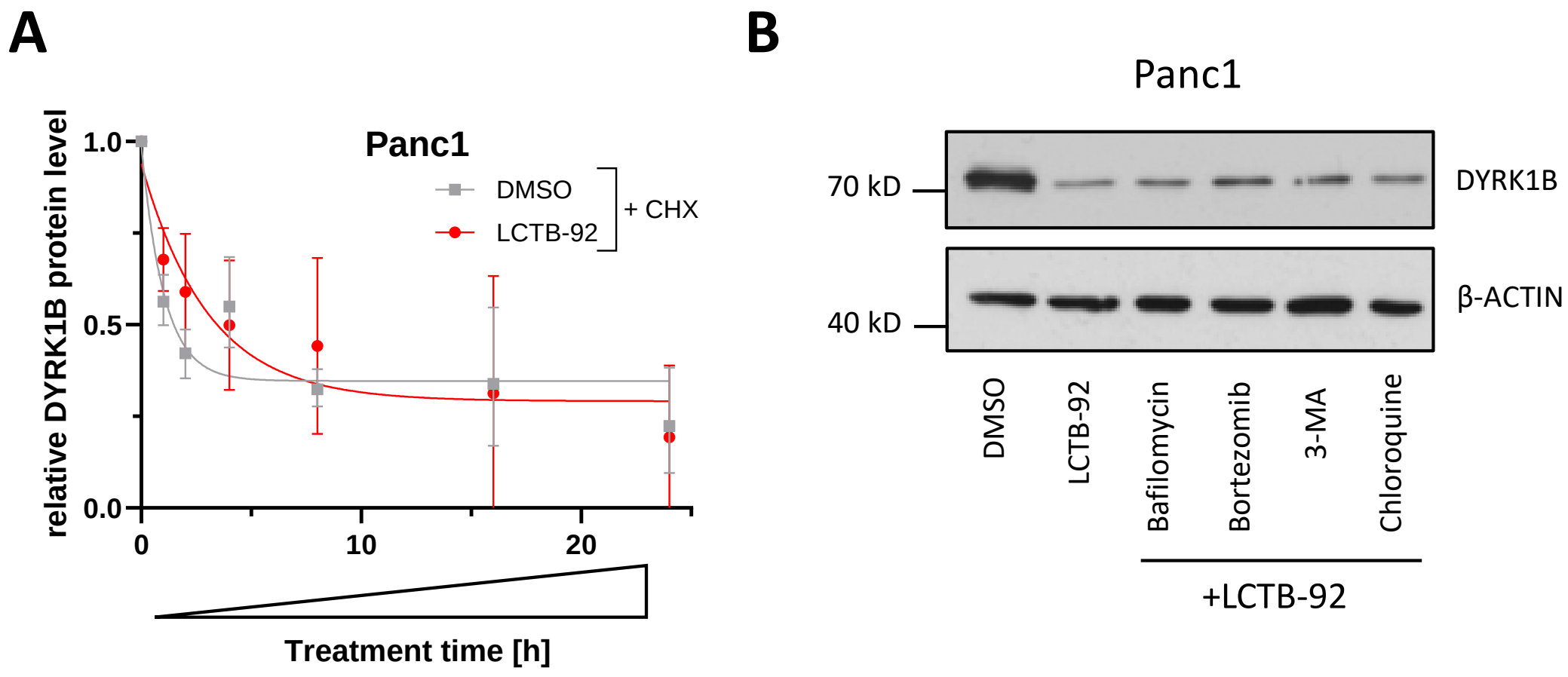

Figure S2. LCTB-92 does not affect DYRK1B protein stability.

A Quantification of DYRK1B protein levels in Panc1 cells upon treatment with LCTB-92 (1 µM) or vehicle (DMSO). Cells were simultaneously treated with Cycloheximide (CHX; 100 µg/ml) for the indicated times. Displayed is the quantification of DYRK1B protein level in relation to β-Actin level, normalized on time point zero. Shown is the mean ± SD of n = 2 independent experiments measured in duplicate. **B** Western blot showing DYRK1B protein levels in 24h-treated Panc1 cells as indicated. Bafilomycin (100 nM), Bortezomib (100 nM), 3-MA (6 mM), and Chloroquine (100 µM). Shown is one representative of n = 2 independent experiments.

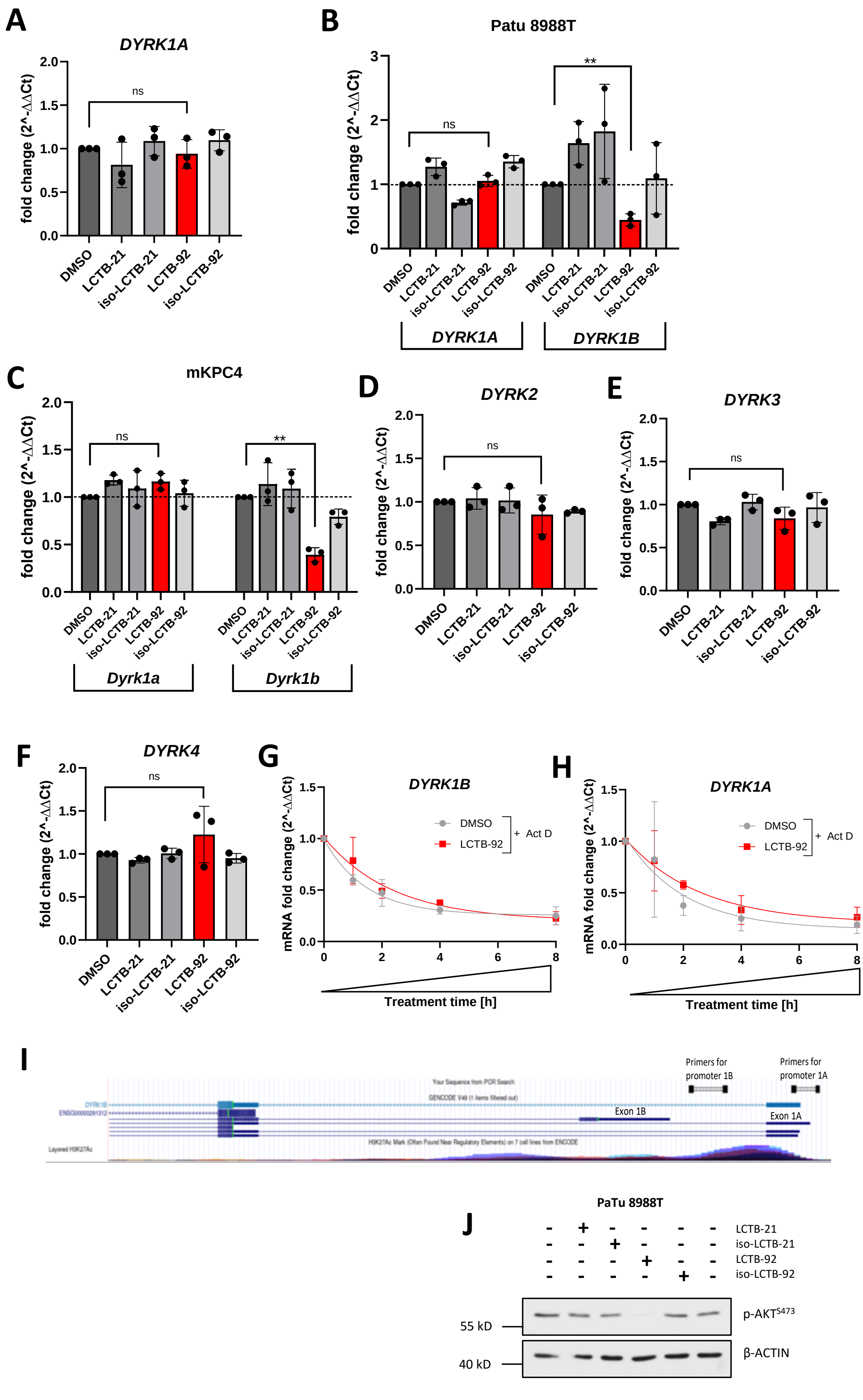

Figure S3. LCTB-92 does not affect *DYRK1B* mRNA stability.

A Quantitative PCR measurement of *DYRK1A* expression in Panc1 cells (24h treatment, 1µM compound). Shown is the mean ± SD of n=3 independent experiments. **B** Quantitative PCR measurement of *DYRK1A* and *DYRK1B* expression in PaTu 8988T cells (24h treatment, 1µM compound). Shown is the mean ± SD of one representative

experiment of n=2 independent experiments (measured in triplicate). **C** Quantitative PCR measurement of *Dyrk1a* and *Dyrk1b* expression in murine mKPC4 cells (24h treatment, 1µM compound). Shown is the mean ± SD of one representative experiment of n=2 independent experiments (measured in triplicate). **D** Quantitative PCR measurement of *DYRK2* expression in Panc1 cells (24h treatment, 1µM compound). Shown is the mean ± SD of n=3 independent experiments. **E** Quantitative PCR measurement of *DYRK3* expression in Panc1 cells (24h treatment, 1µM compound). Shown is the mean ± SD of n=3 independent experiments. **F** Quantitative PCR measurement of *DYRK4* expression in Panc1 cells (24h treatment, 1µM compound). Shown is the mean ± SD of n=3 independent experiments. **G** Quantification of *DYRK1B* mRNA in Panc1 cells treated with LCTB-92 (1 µM) or vehicle control (DMSO) simultaneously with Actinomycin D (10 µM) for the indicated amount of time. Shown is the fold change of *DYRK1B* over time normalized on time point zero. Shown is the mean ± SD of one representative experiment measured in triplicate for n = 2. **H** Quantification of *DYRK1A* mRNA in Panc1 cells treated with LCTB-92 (1 µM) or vehicle control (DMSO) simultaneously with Actinomycin D (10 µM) for the indicated amount of time. Shown is the fold change of *DYRK1A* over time normalized on time point zero. Shown is the mean ± SD of one representative experiment measured in triplicate for n = 2. **I** Schematic depiction of the genomic binding sites of the ChIP-qPCR primer pairs used (Human *DYRK1B*; UCSC Genome Browser). **J** Levels of the mTORC2 target phospho-AKT^{S473} upon the indicated treatments (24h; 1µM compound). Shown is one representative of n=2 independent experiments.

Additional Western blots for verification

WBs showing impact of LCTB-92 on DYRK1B:

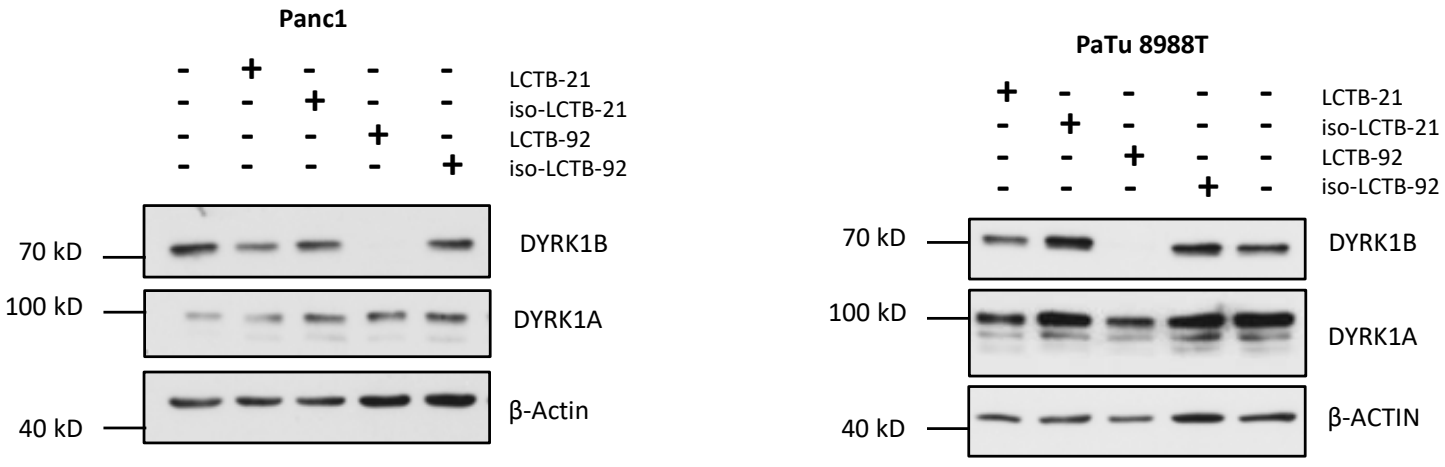

WBs showing dose-dependency of LCTB-92 (vs. LCTB-21):

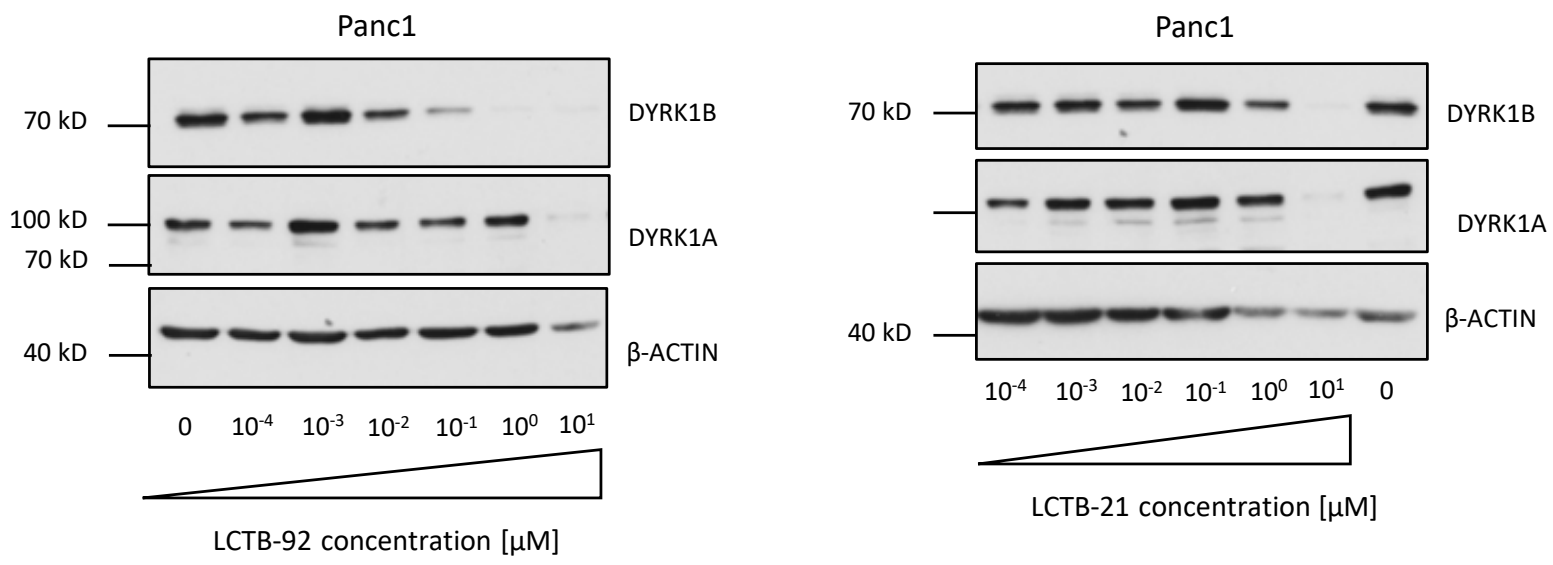

WBs showing time-dependency of LCTB-92 (vs. LCTB-21):

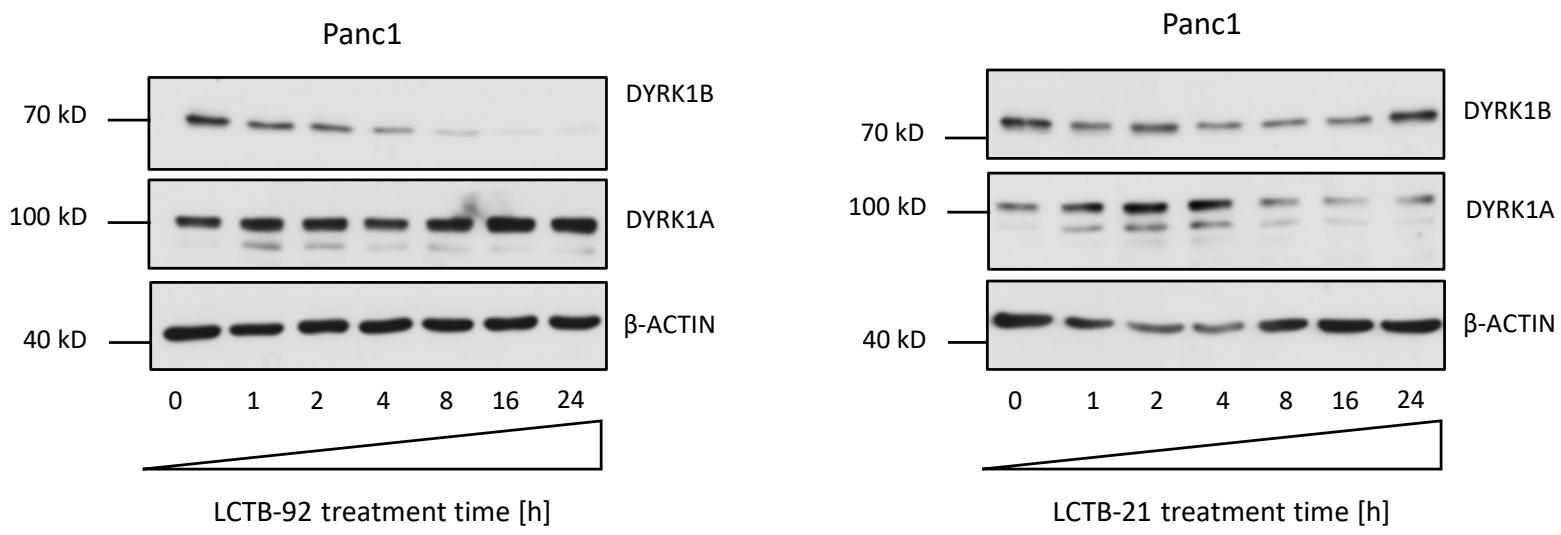

Figure S4. Selected additional Western blots for verification.
